# Complete Genome Sequences of Four *Escherichia coli* ST95 Isolates from Bloodstream Infections

**DOI:** 10.1128/genomeA.01241-15

**Published:** 2015-11-05

**Authors:** Craig M. Stephens, Jeffrey M. Skerker, Manraj S. Sekhon, Adam P. Arkin, Lee W. Riley

**Affiliations:** a Biology Department and Public Health Program, Santa Clara University, Santa Clara, California, USA; b Department of Bioengineering, University of California, Berkeley, California, USA; c Physical Biosciences Division, Lawrence Berkeley National Laboratory, Berkeley, California, USA; d Division of Infectious Diseases and Vaccinology, School of Public Health, University of California, Berkeley, California, USA

## Abstract

Finished genome sequences are presented for four *Escherichia coli* strains isolated from bloodstream infections at San Francisco General Hospital. These strains provide reference sequences for four major *fimH*-identified sublineages within the multilocus sequence type (MLST) ST95 group, and provide insights into pathogenicity and differential antimicrobial susceptibility within this group.

## GENOME ANNOUNCEMENT

The Gram-negative bacterium *Escherichia coli* can be associated with intestinal and extraintestinal infections. “ExPEC” (extraintestinal pathogenic *E. coli*) strains are a major cause of human urinary tract infections, as well as bloodstream infections arising as a consequence of pyelonephritis, a complication of urinary tract infections (1). Antimicrobial resistance among uropathogenic *E. coli* strains is an ongoing clinical challenge (2). Most ExPEC isolates derive from a small number of globally distributed pandemic lineages defined by multilocus sequence typing (MLST) (1). The ST95 MLST group is one of the most frequently observed, and is notable in showing a relatively low frequency of antimicrobial resistance. We report here complete genome sequences from ST95 strains representing four major sublineages associated with distinct alleles of the *fimH* gene (3, 4). These sequences provide references for comparative analysis of drug resistance and host niche adaptation within and beyond the ST95 clade.

The four *E. coli* strains examined here (SF-088, SF-166, SF-173, and SF-468) were isolated from patients with bloodstream infections at San Francisco General Hospital between 2007 and 2010 (4, 5). Sequencing used the Pacific Biosciences (PacBio) RSII platform (Pacific Biosciences, MenloPark, CA, USA). DNA samples were initially purified using a Qiagen DNeasy tissue extraction kit (Qiagen, USA), followed by phenol-chloroform extraction, and sheared to an average fragment size of 20 kb (Diagenode, Liege, Belgium) before conversion into a sequencing library using the SMRTbell Template Prep Kit 1.0 (Pacific Biosciences). Each library was sequenced using P6C4 chemistry on a single-molecule real-time (SMRT) cell with a 240-min collection protocol. Reads were *de novo* assembled and polished using SMRT Analysis v2.3.0 and the HGAP2 or HGAP3 algorithms. For Illumina sequencing, libraries were prepared for 300 bp paired-end reads by incorporating index tags (Wafergen Biosystems). Libraries were sequenced on a MiSeq instrument using V3 chemistry. MiSeq data were used to correct errors in the HGAP assemblies using Pilon (6). Plasmids less than 10 kb in length were assembled solely from MiSeq reads. Annotation of genomes used RAST (7) and the NCBI prokaryotic genome annotation pipeline.

The SF-468 (*fimH-1*) genome includes a 5.14-Mb chromosome and five plasmids (111, 94, 6.7, 4.1, and 1.6 kb). The SF-166 (*fimH-6*) genome is comprised of a 4.91-Mb chromosome and a 114-kb plasmid. The SF-088 (*fimH-9*) genome includes a 5.05-Mb chromosome and three plasmids (150, 5.2, and 3.9 kb). The SF-173 (*fimH-47*) genome is comprised of a 5.06-Mb chromosome and a 93-kb plasmid. The chromosomes are almost completely colinear, with size differences primarily due to prophage. The largest plasmids in each strain are conjugal IncF replicons, and with the exception of SF-166 carry multiple antimicrobial resistance genes. The 94-kb IncI1 plasmid in SF-468 also carries the *blaCTX-M-14* ESBL gene. Other than genes encoding the conjugal machinery in the IncF plasmids, there is relatively little similarity in the remaining plasmid content. More detailed analysis of virulence factors, plasmids, and antibiotic resistance among *E. coli* ST95 strains will be presented in subsequent publications.

### Nucleotide sequence accession numbers.

These sequences have been deposited at DDBJ/EMBL/GenBank under the following accession numbers: SF-468 chromosome and plasmids, CP012625 to CP012630; SF-173 chromosome and plasmid, CP012631 and CP012632; SF-166 chromosome and plasmid, CP012633 and CP012634; SF-088 chromosome and plasmids, CP012635 to CP012638.
